# Effect of trimetazidine on heart rate variability in elderly patients with acute coronary syndrome

**DOI:** 10.12669/pjms.321.8378

**Published:** 2016

**Authors:** Jing Zhang, Shenghu He, Xuefei Wang, Daxin Wang

**Affiliations:** 1 Jing Zhang, Department of Cardiovascular Medicine, Northen Jiangsu People’s Hospital, Medical College of Yangzhou University, Yangzhou, Jiangsu, 225001, China; 2 Shenghu He, Department of Cardiovascular Medicine, Northen Jiangsu People’s Hospital, Medical College of Yangzhou University, Yangzhou, Jiangsu, 225001, China; 3 Xuefei Wang, Department of Cardiovascular Medicine, Northen Jiangsu People’s Hospital, Medical College of Yangzhou University, Yangzhou, Jiangsu, 225001, China; 4 Daxin Wang, Department of Cardiovascular Medicine, Northen Jiangsu People’s Hospital, Medical College of Yangzhou University, Yangzhou, Jiangsu, 225001, China

**Keywords:** Trimetazidine, Elder acute coronary syndrome, Heart rate variability

## Abstract

**Background and Objective:**

Trimetazidine has mainly been used in coronary insufficiency, angina and elderly myocardial infarction. However, the effect of trimetazidine on the efficacy, heart rate variability (HRV) and protection of myocardial ischemia in elderly patients with acute coronary syndrome (ACS) remains unclear. This study aimed to investigate the effect of trimetazidine on the efficacy HRV and protection of myocardial ischemia in patients with ACS.

**Methods:**

One hundred twenty two elderly ACS patients who were above 70 years were chosen and randomly divided into two groups. One group was given conventional therapy, such as aspirin, isosorbide mononitrate and fluvastatin, and the other group was administered trimetazidine in addition to conventional therapy. The treatment period was eight weeks. A PI-2.22B three-channel AECG system was used on every patient for 24 hour dynamic electrocardiogram monitoring and HRV analyses on the first day after admission and eight weeks after treatment. HRV, 24 hour RR intermediate stage standard deviation (SDNN), five minutes average normal cardiac cycle standard deviation in 24 hour (SDANN), 24 hour close together normal cardiac cycle difference value mean square root (rMSSD), the percentage of difference of close together RR intermediate > 50 ms account total RR intermediate (PNN50), high frequency (HF) and low frequency (LF) parameters of patients were observed before and after treatment.

**Results:**

The SDNN, SDANN, rMSSD, PNN50 and HF parameters significantly increased compared with the conventional treatment group (all P < 0.05). LF and LF/HF were significantly decreased in the trimetazidine treatment group compared with those in the conventional treatment group (all P < 0.05).

**Conclusion:**

Trimetazidine improves HRV of elderly ACS patients and reduces cardiovascular events.

## INTRODUCTION

Acute coronary syndrome (ACS) includes unstable angina (UA) and acute myocardial infarction (AMI). Cardiovascular diseases are currently the most common cause of death worldwide. ACS is a popular cardiovascular disease with poor prognosis, and many patients die from abnormal heart rate variability (HRV).^1^,^2^ Although a great deal of advanced therapies can be used to relieve the coherent morbidity and mortality in developed countries, a large amount of patients in developing countries still suffer from the threat of ACS.^3^ Therefore, drugs for the improvement of ACS are being introduced.

Trimetazidine, with its chemical name trimetazidine, 3-KAT depressantia, is an anti-heart muscle ischemia drug that has mainly been used in coronary insufficiency, angina and elderly myocardial infarction. Previous studies have reported that trimetazidine can accommodate autonomic nerve function, recover heart function, improve the condition of heart muscle ischemia and increase the stability of coronary heart disease (CHD).^4^-^7^ Moreover, other studies showed that trimetazidine can reduce tissue damage caused by oxidation and tissue fibration,^8^-^10^ as well as decrease intracellular acidosis caused by the lipometabolism and anaerobic metabolism of ischemic cells.^11^ Trimetazidine is also believed to reduce restenosis after stenting and occurrence of other cardiovascular events.^12^,^13^

In addition, trimetazidine can ameliorate the prognosis of ACS by interfering with HRV.^14^ HRV with no traumatic occlusion is a novel method to quantitatively assess heart autonomic nervous system function and diagnose CHD.^15^ This technique has been used in many cardiovascular diseases. Abnormal HRV in patients with USTEMI can lead to a high mortality.^16^ Besides, some reports showed that abnormal HRV is one of the causes of the increase in the morbidity and mortality of cardiovascular diseases.^17^ We observed the curative effect and changes before and after medication with trimetazidine and conventional therapy to 122 elderly ACS patients from January 2013 to December 2014.

## METHODS

One hundred twenty two elderly patients with ACS (age: 70 years and above) were selected in this study from January 2013 to December 2014. The inclusion criteria were patients with UA and non-AMI (consistent with ACC/AHA<<UA and AMI treatment guidelines>>(2000) in the UA and AMI diagnostic criteria), representative chest pain for 10 minutes or above 24 hour prior to hospitalisation, electrocardiogram change, increase in heart muscle necrosis marker (phosphocreatine kinase isoenzyme (CK-MB) and treponin) and 70 years old or above. The average contractive pressure of patients was 132±17 mmHg, and the average diastolic pressure of patients was 83±10 mmHg. All 122 patients were divided into the trimetazidine group and conventional therapy group. The trimetazidine group comprised 58 patients (38 UA cases (65.5%) and 20 AMI cases (52.6%)). In this group, 31 were men (53.4%) and 27 were women (46.6%). The average age in the trimetazidine group was 78±7 years. The conventional therapy group comprised 64 patients (36 UA cases (56.3%) and 28 AMI cases (43.8%)). In this group, 30 were men (46.9%) and 34 were women (53.1%). The average age in the conventional therapy group was 79±6 years. No obvious differences in sex, age and concomitant disease were found between the two groups. The study was approved by the Medical Ethics Council of Medical College of Yangzhou University.

### Methods

The patients in the conventional therapy group were given aspirin, isosorbide mononitrate and fluvastatin for eight weeks. The patients in the trimetazidine group were given aspirin, isosorbide mononitrate and fluvastatin for eight weeks, with the addition of trimetazidine (20 mg). Trinitrate was used by the hypoglossus to relieve symptoms if chest pain remained obvious during medication.

### Observation indicatrix

A PI-2.22B three-channel AECG system (PI Company, USA) was used for 24 hour ambulatory blood pressure monitoring and HRV analyses of the patient on the first day after admission and eight weeks after treatment. HRV was deployed by time domain and frequency domain. Time domain: (1) SDNN: 24 hour RR intermediate stage standard deviation; (2) SDANN: five minutes average normal cardiac cycle standard deviation in 24 hour; (3) rMSSD: 24 h close together normal cardiac cycle difference value mean square root; (4) PNN50: the percentage of difference of close together RR intermediate > 50 ms account total RR intermediate. Frequency domain: low frequency power (LF), high frequency power (HF), ratios of low frequency power/ high frequency power (LF/HF).

### Statistical analysis

Data are presented as the mean ± standard deviation (SD). A P value of 0.05 or less was considered statistically significant. Differences before and after medication were evaluated using matched-pairs t-test. Changes in the data between the two groups before and after medication were analysed by t-test.

## RESULTS

The clinical symptoms of patients with UA and NSTEMI were relieved, ST segment depression was improved and the extent of myocardial ischemia was significantly reduced in the trimetazidine treatment group compared with those in the conventional therapy group. Table-I. The clinical symptoms of patients in the trimetazidine treatment group were clearly better than those in the conventional treatment group. HRV time domain and frequency domain significantly improved in the trimetazidine treatment group compared with those in the conventional therapy group.

**Table-I:** HRV change before and after therapy in two groups patients.

	Trimetazidine group (58 cases)		Conventional therapy group (64 cases)	

	Before medication	After medication	Before medication	After medication
SDNN (ms)	155±27	235±41^*Δ^	151±26	155±22
SDANN (ms)	142±22	156±27^*Δ^	141±26	145±23
rMSSD (ms)	97±32	145±50^*Δ^	99±31	120±58*
PNN50 (%)	20.5±17.8	27.7±22.7*	20.0±17.7	22.6±20.4
LF (ms^2^)	572±95	426±91^*Δ^	568±88	558±97
HF (ms^2^)	110±42	132±32*	107±25	111±26
LF/HF	2.47±0.68	1.36±0.58^*Δ^	2.54±0.77	2.41±0.67

* P<0.05, compared with before medication in the trimetazidine group;

Δ P<0.05, compare with after medicatine in conventional therapy group.

## DISCUSSION

ACS, which includes unstable angina, non-ST-elevation myocardial infarction and ST-segment elevation myocardial infarction, is a syndrome of a group of CHDs. This group includes coronary atherosclerosis plaque rupture, haemorrhage, thrombosis, coronary spasm, severe stenosis, trauma and mezzanine as the basic pathophysiological characteristics, with the common characteristics of necrosis or acute myocardial ischemia caused by increasing myocardial consumption of oxygen, leading to hyperpyrexia, severe anaemia and hyperthyroidism. At present, the treatment of ACS includes anticoagulant therapy, inhibiting platelet aggregation, vasodilators, anti-myocardial ischemia, reducing blood fat, stabilising plaque, improving myocardial remodelling, preventing sudden death and reduction of risk factors.

The mechanism of trimetazidine affecting the metabolism of cardiac ischemic cells is to inhibit the oxidation of fatty acid phosphate by activating pyruvate dehydrogenase, transfer oxidative substrate from fatty acids to glucose, promote the use of glucose, improve the energy efficiency of cardiac cells and lower the side effects of fatty acid oxidation. In addition, trimetazidine can interfere with phospholipid metabolism, which remarkably improves 3-inositol utilisation and enhances the conversion rates of membrane phospholipids.^18^ The stability of its membrane structure is maintained under hypoxia. The dose of trimetazidine does not affect hemodynamic stability, heart rate, blood pressure and speed-pressure multiplication, and it exerts no negative inotropic effect. The results showed that trimetazidine could relieve the clinical symptoms of patients with UA and NSTEMI, improve ST segment depression and reduce the extent of myocardial ischemia. In our study, the clinical symptoms of patients with UA and NSTEMI were obviously relieved in the trimetazidine treatment group compared with those in the conventional therapy group.

HRV is an indicator that has been raised recently to forecast cardiovascular events and sudden cardiac death. All indicators about HRV were defined by special reports published in Circulation in 1996. The time–domain indicators SDNN, which mainly reflects parasympathetic and sympathetic tension; rMSSD; PNN50, which reflects vagal tone and is associated with rapid changes in heart rate; and PNN50 decreased with the reduction in vagal tone. SDANN reflects sympathetic tension, which was associated with the slow changes in heart rate. The value of SDANN increased with increasing sympathetic nervous tension. HF is associated with vagus nerve activity, and LF is associated with sympathetic nervous activity. LF/HF is an indicator that comprehensively reflects the balance of the vagus nerve and sympathetic nervous system. Excitement of the sympathetic nervous system can reduce the ventricular fibrillation domain, whereas that of the vagus nerve can improve the ventricular fibrillation domain. Once the regulation of the autonomic nerve to the heart is low, particularly under low vagus activity, the electrical stability of cardiac cells decreases and the ventricular fibrillation domain weakens, thereby triggering fatal arrhythmia.^19^ Therefore, measuring HRV has significant value for assessing nervous tension in elderly patients with ACS. Moreover, trimetazidine can improve the prognosis of ACS by improving HRV.

Topal E et al. treated 48 patients of slow coronary blood flow with trimetazidine.^20^ They also detected the HRV parameters, NO and ET-1. They reported that the drug significantly improves HRV parameters and has good correlation with NO and ET-1. Ulgen MS et al. also found that trimetazidine can improve HRV in patients with myocardial infarction and reduce the incidence of malignant arrhythmia, such as ventricular tachycardia and ventricular fibrillation.^21^ Our research showed that HRV time domain and frequency domain in elderly patients with ACS in the trimetazidine group significantly improved compared with those in the conventional therapy group. Our results have demonstrated that trimetazidine could maintain the balance of autonomic nerves and regulate the heart by improving the metabolism of ischemia myocardial cells in elderly patients with ACS. The underlying mechanism was possibly related to reducing sympathetic nervous tension resulting from the feedback of ischemia, heightening the ventricular fibrillation domain and decreasing the incidence of malignant arrhythmia. However, its relation to nervous tension remains unclear, and large trials in the future are necessary.
